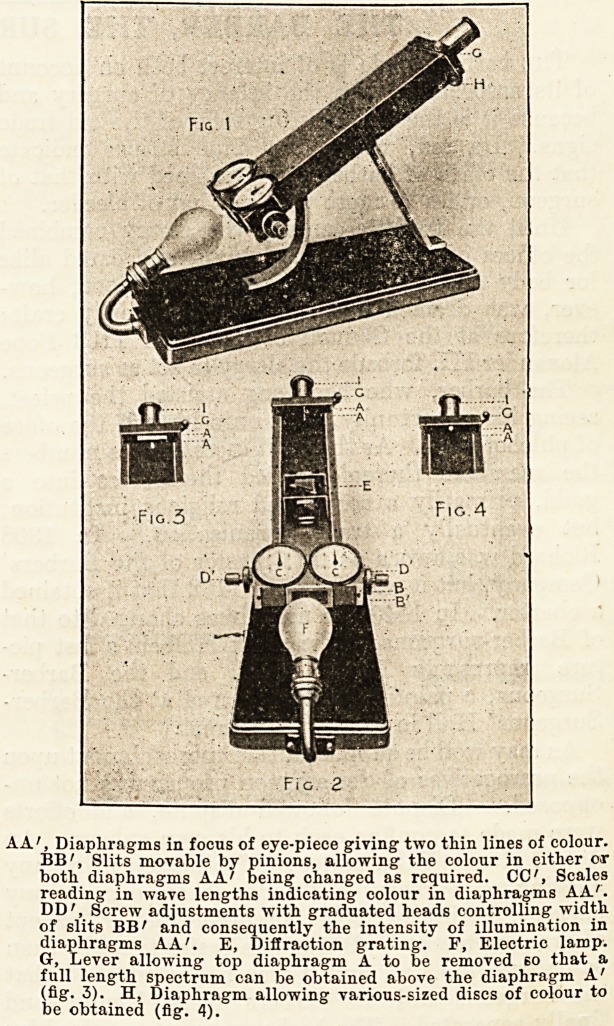# A Spectroscopic Test of Colour Vision

**Published:** 1908-10-31

**Authors:** A Maitland Ramsay

**Affiliations:** Surgeon, Ophthalmic Institute, Glasgow Royal Infirmary


### Ophthalmology.

### A SPECTROSCOPIC TEST OF COLOUR VISION

By A. MAITLAND RAMSAY, M.D., Surgeon, Ophthalmic Institute, Glasgow Royal Infirmary.

I have recently had constructed an instrument by means of which the natural colours of the spectrum are used as a test of colour vision. It combines in itself the matching test as ordinarily carried out by Holmgren's wools, and the lantern test so strongly advocated by Mr. Edridge Green and others. In all scientific investigations of the colour sense the spectrum is always employed, but up till now the difficulties of its application have been a hindrance to its use in ordinary clinical work. It was to try to overcome these obstacles that several years ago I began to experiment with a diffraction grating, and after many trials the instrument I am at present using has been devised.

Every colour test must be absolutely reliable, easily understood, and so readily applied that a large number of persons can be tested in a short time. All these conditions are fulfilled in the spectroscopic colour test, which also possesses the additional advantage that in the examination the pure colours of the spectrum are employed instead of hues artificially produced. Moreover, the instrument is convenient in size, light and portable. It is simple in construction, and has no part liable to get out of order or to be injured by use or by ordinary change of temperature, so that the apparatus could be readily used on board modern steamships or in railway works; wherever, indeed, an electric current can be obtained. The lamp can be connected with the electric main by an ordinary plug, care being, of course, taken that the lamp is of a voltage suitable for the strength of the current. The instrument consists of a rectangular brass box, carefully blackened on the inside, and mounted on a double metal support resting on a wooden base. It is so constructed that two spectra of considerable dispersion are formed in the focus of an eye-piece, and by an arrangement of shutters and screws any portion of the spectrum can be brought into view as desired. The movement of the spectra is indicated by the movements of the indices on two dials placed at the same end of the instrument as the lamp, but outside the box, and so situated that they cannot be read by the patient. At the other end of the test is a rotatory diaphragm perforated by three circular apertures of different size, which, when the upper shutter is displaced, can be turned into position. This arrangement permits only a small circular spot of colour to be seen, and enables the correct naming of colours to be tested as well as the correct matching. The illumination of the colours is controlled by graduated adjusting screws; in this way the brilliance of the colour in the spectrum can be diminished or increased at will. A double image prism can be adjusted over the eye-piece of the instrument.

The apparatus is used in the following way: The examiner, having by means of the right hand screw, underneath the lower end of the box, brought any portion of the spectrum he pleases opposite the lower slit, as a test the patient is seated in front of the eye-piece, and asked to look through the lens and to turn the left-hand screw underneath till the colour seen through the upper slit is exactly the same as that visible through the lower. The examiner can tell what the patient is doing by watching the movements of the index on the left-hand dial; and if he write down the registered index figures on both dials he can keep a written

AA′, Diaphragms in focus of eye-piece giving two thin lines of colour. BB′, Slits movable by pinions, allowing the colour in either or both diaphragms AA′ being changed as required. CC′, Scales reading in wave lengths indicating colour in diaphragms AA′. DD′, Screw adjustments with graduated heads controlling width of slits BB′ and consequently the intensity of illumination in diaphragms AA′. E, Diffraction grating. F, Electric lamp. G, Lever allowing top diaphragm A to be removed so that a full length spectrum can be obtained above the diaphragm A′ (fig. 3). H, Diaphragm allowing various-sized discs of colour to be obtained (fig. 4).

record of the examination. To begin with, the whole upper spectrum may be exposed to view, this being done, the person whose colour-sense is being tested is asked to bring the corresponding portion of it immediately above that shown in the lower slit. The upper diaphragm, suggested to me by Edridge Green, is then put in position, and the patient can be asked not only to match the colour seen in the lower slit, but also to turn the left-hand screw underneath, and to say at once whenever he detects the slightest change in colour in either direction. The test colours can be varied at will, and each eye ought to be tried separately.

Lastly, the upper slit diaphragm should be displaced, and the rotatory one brought into use. A spot of colour is now in the line of central vision and can be varied as the examiner pleases, the patient being asked to name the colours, just as would be done if the lantern test were being employed. The instrument may thus be used, with very trustworthy results, to detect colour scotoma in toxic amblyopia (acquired colour-blindness), the patient being unable to recognise the colour (mostly red or green) in the central spot. If the double-image prism be placed over the eye-piece the test becomes still more delicate; for the patient, while he cannot see the central stationary spot, can, by peripheral vision, see the eccentric one as it revolves when the prism is rotated. By diminishing the amount of light and noting the reading of the graduated screw-heads, it is possible to form an approximate estimate of the acuity of the patient's colour-light perception; for example, how quickly and readily the patient could recognise the colour of a railway signal or of a ship's light in foggy weather? The instrument is made by John Trotter, optician, 40 Gordon Street, Glasgow.

## Figures and Tables

**Figure f1:**